# Exploring Mechanisms behind Migration’s Impact on Protein Intake of Left-Behind Household Members: A Panel Analysis from China

**DOI:** 10.3390/ijerph21060652

**Published:** 2024-05-21

**Authors:** Xueting Pan, Jiaqi Huang

**Affiliations:** 1 School of Urban and Regional Science, Shanghai University of Finance and Economics, Shanghai 200433, China; yuan2123@126.com; 2 Agricultural Information Institute, Chinese Academy of Agricultural Sciences, Beijing 100081, China

**Keywords:** protein intake, migration, agricultural production, family size, China

## Abstract

Malnutrition remains a critical global health challenge, especially in rural areas, where it significantly impacts the health and economic stability of households. This study explores (1) the relationship between labor migration and dietary protein intake in households remaining in economically disadvantaged rural regions and (2) the influence of remittance income, farm earnings, self-produced food, and changes in family size due to migration on their dietary protein. Panel data were collected through a three-wave household survey of 1368 rural households across six counties in the provinces of Guizhou, Yunnan, and Shaanxi during 2012, 2015, and 2018. Employing a two-way fixed effects model, we found that labor migration positively affects the protein consumption of families left behind. The mediated effects model indicated that decreases in family size had the most significant impact on protein intake, with a value of 8.714, accounting for 0.729 of the total effect; followed by the mediating effect through crop income, at 2.579, representing 0.216 of the total effect; and livestock income, at 0.772, contributing 0.073 of the total effect. However, the mediating effects of remittance income and self-production were found to be insignificant. In conclusion, our study found that migration improves protein intake primarily through increased crop and livestock production and decreased family size. These results highlight the critical role of family structure and farm productivity in enhancing the nutrition of families affected by labor migration, offering valuable insights for policymakers.

## 1. Introduction

Nutrition in rural areas has long been a global challenge [1], as malnutrition adversely affects both the attainment and maintenance of health and hampers the accumulation of human capital. It also restricts individuals’ ability to increase their incomes, thus exacerbating the vicious cycle of poverty in these regions [2,3]. Influenced by low disposable incomes and unhealthy dietary habits, rural residents in impoverished areas often opt for cheaper, more energy-dense foods that are high in carbohydrates, salt, and fats rather than healthier options rich in proteins and vitamins [4,5]. This preference makes them more susceptible to malnutrition.

Addressing malnutrition effectively requires tackling the root causes of poverty and low incomes. Increasing employment stands as the most effective and direct method to enhance income levels and alleviate poverty [6]. Furthermore, it is essential that impoverished populations use their increased incomes to improve their diets. Enhancing home environments [7] and improving access to affordable, nutritious food [8] are crucial steps in boosting their nutritional intake.

In rural China, migrant labor has emerged as a key strategy for poverty alleviation and income generation [9]. Rural-urban migration and changes in the occupational patterns of the rural labor force have dramatically transformed the economic and social structures of these areas [10], affecting the nutritional intake of the family members left behind. Currently, there is an ongoing debate regarding the impact of migrant labor on the nutritional status of these rural residents. Some studies suggest that the migration of rural labor increases the risk of nutritional insecurity among vulnerable family members, such as children and the elderly [11,12]. Conversely, other research indicates that migrant labor has actually improved the nutritional status of rural residents [13,14]. To date, relatively few studies have explored the mechanisms influencing labor migration and have focused primarily on remittances. However, this narrow focus may not fully explain the varied dynamics observed over time and across geographic regions. More studies have examined the factors influencing the nutritional intake of rural residents, such as income [15], production [16], household dynamics [17], geographic differences [18], and environmental influences [19]; yet, these factors have not been sufficiently linked to migrant labor. Based on the existing literature, we hypothesize that migrants influence protein intake in three key ways: firstly, by increasing disposable income through remittances and agricultural income [20]; secondly, by consuming home-grown agricultural products [21]; and thirdly, by altering household composition [22].

This paper contributes to the ongoing discussion about the impact of migration on the nutritional intake of rural households in China’s less developed regions. It specifically seeks to answer the following questions: Does migrant labor enhance the protein intake of rural residents? What are the primary pathways through which this impact occurs? Exploring these issues is highly relevant in the context of China’s new era. As the world’s largest developing country, having achieved significant progress in poverty alleviation, it is crucial to understand the effects of rural migrant labor on the protein intake of left-behind families in less developed areas. This study provides valuable insights for policymakers in designing and implementing strategies for poverty reduction, decreasing the incidence of poverty, and promoting the development of rural health and the economy.

## 2. Data and Methods

### 2.1. Data

#### 2.1.1. Study Sample

The data in this paper come from a panel database developed from the Rural China Poverty and Food Security Household Longitudinal Survey (RCPFSHS) in poor counties in China [23]. The data used in this study cover tracking survey data from 1368 farm households in 2012, 2015, and 2018 in six of the most food-insecure poor counties: Zhen’an and Luonan counties in Shaanxi Province, Wuding and Huize counties in Yunnan Province, and Panzhou and Zheng’an counties in Guizhou Province. The selection of sample counties in the study was based on A Report on the Status of China’s Food Security [24]. The report analyzed the food security situation of 592 nationally defined key poverty counties in the year of 2010 using county-level statistics, mainly in terms of food supply capacity, food availability, food utilization conditions, food consumption and nutrition, and vulnerability of food supply in the poor counties, and classified the 592 counties into three types, of which 271 counties were the most food-insecure. The six sample counties selected for this paper were chosen from the 271 food-insecure counties, taking into account the willingness and basis of cooperation, as well as the availability of relevant data. 

All six counties are in remote mountain areas, and generally face great economic and social challenges, such as more frequent natural disasters, less developed markets, and weaker infrastructures, which place local residents in an inherently disadvantaged position. 

#### 2.1.2. Measurements and Data Collection Plan

The survey adopts a combination of probability proportional to size sampling and random sampling for sampling [23]. In the first stage, 19 villages were sampled according to the population proportionate sampling (PPS) method in accordance with the number of people in each village in each county, and the more populous the village, the higher the probability of being sampled. In the second stage, a random sampling method was used to randomly select 12 farm households in each sample village. In this way, 228 households were sampled from 19 villages in each county, totaling 1368 households from 114 villages in the six counties.

The study performed follow-up surveys on the same villages and the same farm households in 2012, 2015, and 2018. It should be noted in particular that, in the 2018 revisit, if the investigators encountered a revisited farmer who was not at home during the household survey, they would replace the revisited farmer with another farmer from the same village. Therefore, the total number of sample farm households in 2018 was 1371. 

To ensure data quality, the data survey was divided into three phases: the field survey phase, the pre-data-entry phase, and the data cleaning phase. The field survey phase required the enumerator, team leader, and other team members to check the completeness of the questionnaire. The pre-data-entry phase involved another review of all paper questionnaires, while the data cleaning phase entailed using data analysis software, such as STATA/SE 17.0. and SPSS V 29.0, to logically check the survey data, identify missing values, detect outliers, and perform other necessary checks.

The questionnaire covered basic household information, education, migrant labor and remittances, housing and production conditions, household property and financial status, agriculture, livelihoods, expenditures, food sources and consumption, shocks, and coping strategies. To obtain more stable and comprehensive food consumption data, the questionnaire used the past 30 days as the recall period and recorded household consumption of 167 specific food items, categorized into 13 food groups: cereals, legumes, tubers, vegetables, fruits, aquaculture products, meats, eggs, dairy products, fats and oils, condiments, beverages, and others. These food groups were used to calculate the protein intake of rural households. The primary aim of this study is to explore the mediating factors between migrant labor and the protein intake of household members left behind, thereby providing insights into how migration influences rural family nutrition and suggesting potential interventions to mitigate any negative impacts. 

### 2.2. Econometric Approach

#### 2.2.1. Total Effects of Migration on Protein Intake

We used the panel data of households to conduct a two-way fixed effects model to estimate the total effect of rural household labor migration on the protein intake of left-behind family members. The unit of analysis is the household, and we controlled for household demographics and natural characteristics [25].

(1) $$P{rotein}_{i,t}=\alpha_{0}+\beta_{0}M{ig}_{it}+\theta_{0}X_{i,t}+\mu_{i}+\tau_{t}+\varepsilon_{i,t}$$

where $P{rotein}_{i,t}$ denotes the per capita protein intake for household i in year t. $M{ig}_{it}$ is a binary indicator, taking the value 1 if household i engaged in labor migration. The variables $X_{i,t}$ represent time-variant observable factors at the household and county levels. $\mu_{i}$ captures county-level fixed effects, accounting for time-invariant unobserved heterogeneity. $\tau_{t}$ are year dummies representing time trends and shocks, and $\varepsilon_{i,t}$ represents the standard error adjusted for clustering at the household level.

#### 2.2.2. Direct and Indirect Mediated Effects 

To analyze the pathways through which migration affect protein intake, we estimated the following mediation model at household level using structural equation modeling, as suggested by Baron and Kenny [26].

(2) $${Mediator}_{i,t}=\alpha_{1}+\beta_{1}M{ig}_{it}+\theta_{1}X_{i,t}+{\mu ’}_{i}+{\tau ’}_{t}+{\varepsilon ’}_{i,t}$$

(3) $$P{rotein}_{i,t}=\alpha_{2}+\beta_{2}M{ig}_{it}+\gamma_{2}{Mediator}_{i,t}+\theta_{2}X_{i,t}+{\mu^{\prime \prime }}_{i}+{\tau^{\prime \prime }}_{t}+{\varepsilon \prime \prime }_{i,t}$$

where ${Mediator}_{i,t}$ represents the factors influencing protein intake through migration, encompassing three mediating components: income factors (remittance, crop income, livestock income), the self-production factor, and the family size factor. These indicators operate as separate variables in the model that affect the outcome variables. Specific definitions of the indicators are shown in Table 1.

The structural equation model estimates mediation effects by decomposing the total effect ${(\beta }_{0})$ into an indirect mediated effect $\left(\beta_{1}\times \gamma_{2}\right)$ and direct effect ${(\beta }_{2})$. The indirect mediated effect provides an explanation of how migration operates through the mediators under analysis, shedding light on specific pathways. On the other hand, the direct effect encompasses all other channels through which protein affordability and accessibility are enhanced. 

**Figure 1:**
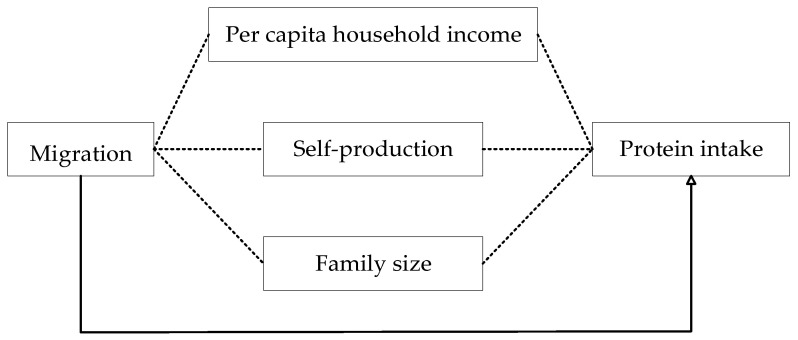
Direct and indirect mediated effects.

Figure 1 delineates the direct effect (depicted by solid arrows) and distinguishes it from the indirect effects, wherein the dashed lines illustrate the mediation effects mediated by per capita household income, self-production, and family size.

### 2.3. Econometric Approach

#### 2.3.1. Outcome Variables 

The protein intake of rural household members is measured in grams per standard person per day, calculated through a multi-step process. Initially, utilizing the protein content per unit of various foods as outlined in the China Food Composition Table, the monthly consumption of specific food items is converted into the total protein intake for the surveyed month (30 days) and then further adjusted to derive the daily protein intake per household. Subsequently, acknowledging individual variations in protein requirements based on age and gender differences, this study adheres to the Chinese Dietary Reference Intakes (DRIs) [27] to standardize each household member into equivalent standard persons. The total standard persons in the household are then aggregated. Finally, the daily protein intake per household is divided by the total standard persons to obtain the daily protein intake per standard person for that household.

#### 2.3.2. Key Explanatory Variables 

The explanatory variables include labor migration and migration rate. Labor migration is operationally defined as the phenomenon wherein workers, whose *hukou* (a system of household registration used in mainland China) remains registered in rural areas, depart from their designated residence to participate in labor across both agricultural and non-agricultural sectors, thereby earning wage income. The migration variable employed in the analysis is a binary variable, indicating whether a household has members engaged in labor migration, with a value of 1 indicating engagement and 0 denoting non-engagement. The migration rate is calculated as the proportion of household members engaged in labor migration relative to the total household population.

#### 2.3.3. Mediating Variables 

Remittance income is money and items brought home by family members who engaged in labor migration. Crop income includes earnings derived from grain cultivation and economic crop cultivation. Livestock income emanates from animal husbandry within household management, including income generated by raising poultry and livestock, such as pigs, cows, sheep, as well as income from fish farming and other aquatic products. Self-production refers to the amount of consumed food originating from self-production. Family size denotes the population size of the left-behind family members.

#### 2.3.4. Control Variables 

In addition to the key variables mentioned above, this study also controls for household head characteristics (gender, age, and schooling), household characteristics (per capita annual household income, the proportion of young children aged (0–5), the proportion of school-aged children (aged 5–14), the proportion of elderly individuals, household wealth index, household indebtedness, distance to the nearest market, and land cultivated), and natural characteristics (natural shocks and other shocks).

The definitions and assignments of all variables utilized in this study are presented in Table 1.

**Table 1:** The definition and assignments of variables.

Variables	Definition and Assignments
Outcome variables	
Protein intake of rural household members	The daily protein intake per equivalent person = ∑(amount of each food consumed in the last 30 days) × unit protein content of each food)/(number of equivalent person × 30) [27] Unit: grams
Key explanatory variables	
Migration	Whether a household has members engaged in labor migration, Yes = 1, No = 0
Migration rate	The ratio of the number of household members engaged in labor migration to the total household population
Mediating variables	
Remittance	The natural logarithm of the per capita remittance income (unit: yuan) of left-behind family members
Crop income	The natural logarithm of per capita crop income (unit: yuan) of left-behind family members
Livestock income	The natural logarithm of per capita livestock income (unit: yuan) of left-behind family members
Self-production	The natural logarithm of the amount of consumed food (unit: kilograms) originating from self-production
Family size	The population size of the left-behind family members, unit: people
Control variables	
Gender of household head	Male = 1, Female = 0
Age of household head	unit: year
Schooling of household head	Educational years of the household head, unit: year
Annual per capita income	The natural logarithm of per capita annual household income (unit: yuan) after deducting remittances from labor migration
Proportion of young children	The proportion of young children (aged 0–5)
Proportion of school-aged children	The proportion of school-aged children (aged 5–14)
Proportion of the elder	The proportion of elderly individuals aged above 65
Household wealth index	The index measures the affluence level of a household, encompassing a comprehensive range of aspects including housing conditions, durable goods, productive assets, transportation, and access to water and electricity [23]
Household indebtedness	Whether the household has debt, Yes = 1, No = 0
Distance market	The distance to the nearest market or marketplace (unit: kilometers)
Land cultivated	The natural logarithm of household cultivated land area (unit: mu)
Natural shock	Whether experiencing a natural disaster, Yes = 1, No = 0.
Other shock	Whether experiencing other disasters, Yes = 1, No = 0.

## 3. Results

### 3.1. Summary Statistics

All analyses were conducted in Stata/SE 17.0.

Our sample comprises 1368 households in 2012 and 2015 and 1371 households in 2018. Table 2 provides summary statistics for household-level core indicators (panel A), mediated indicators (panel B), and the set of control variables (panel C). Panel A shows that protein intake, migration, and migration rate have the same trend, decreasing in 2015 and increasing in 2018. Panel B shows that per capital remittance increases each year while per capital crop income and livestock income drop. The level of self-production experiences a significate decrease from 2012 to 2018. The variation in family size is minimal, with an average of 3.4 individuals residing in rural households.

In panel C, nearly 90% of the sample households were headed by men. The average education level of the sampled household heads was approximately 6.4 school years. In terms of household characteristics, the proportion of children and the proportion of elderly individuals have increased, while the proportion of school-aged children has declined. Meanwhile, per capita income and household wealth index have increased over time, indirectly suggesting an enhancement in household income and wealth due to the involvement of migrant workers. The percentage of households with debt in the years 2012, 2015, and 2018 stood at approximately 61%, 60%, and 58%, respectively. There is a consistent decrease in the distance of households to the nearest market, indicating ongoing improvements in rural infrastructure. Notably, there is an observable fluctuation in the cultivated land area of households over the given period.

In 2012, approximately 84% and 70% of the sample households experienced natural shocks and other shocks. By 2015, the figures dropped to about 62% for natural shocks and 51% for other shocks. In 2018, further reductions were observed, with 43% of households encountering natural shocks and 26% experiencing other shocks.

### 3.2. Results of the Total Effects of Labor Migration 

**Figure 2:**
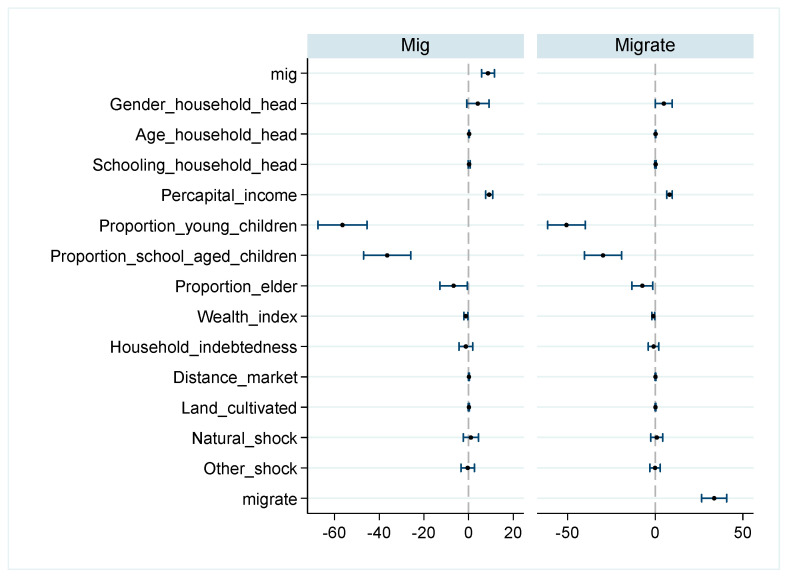
The effects of per capita protein of rural family.

Table 3 and Figure 2 provide the results of the total effects of migration on per capita protein intake, employing the indicators of migration engagement (column 1) and migration rate (column 2), respectively. The results show that migration has significant positive effects on per capita protein intake, evidenced by migration (*Coef.* = 8.701, *p* < 0.01) and migration rate (*Coef.* = 33.638, *p* < 0.01).

Within household head characteristics, education and gender exhibit no significant impact (*p* > 0.1) on protein intake, while age (*Mig Coef.* = 0.261, *p* < 0.01, *Migrate Coef.* = 0.204, *p* < 0.05) exerts a positive influence on the protein intake of family members. Regarding household characteristics, per capita income (*Mig Coef.* = 9.223, *p* < 0.01, *Migrate Coef.* = 8.184, *p* < 0.05) significantly contributes to protein intake in the family, whereas households with young children (*Mig Coef.* = −56.445, *p* < 0.01, *Migrate Coef.* = −50.619, *p* < 0.01) and school-aged children (*Mig Coef.* = −36.416, *p* < 0.01, *Migrate Coef.* = −29.766, *p* < 0.01) have a negative impact. The remaining control variables show no significant effects. 

### 3.3. Mediation Results

Table 4 presents the causal mediation analysis structure for the effects of migration on protein intake. In Table 4, column (1) presents the impact of migration on the intermediate variables, corresponding to $\beta_{1}$ in Model (2). Core explanatory variables related to migrant work, namely crop income and livestock income, exhibit significantly positive coefficients of 0.404 and 0.180, respectively. The variable of migration for family size reveals a significantly negative coefficient of −0.835. Remittance and self-production variables do not show statistical significance. 

Column (2) presents the effects of per capita household income, self-production, and family size on protein intake, corresponding to $\gamma_{2}$ in Model (3), and all variables are significant. Column (3) represents the direct effects of migration on protein intake, corresponding to $\beta_{2}$ in Model (3). The direct effects of the models with intermediate variables as crop income, livestock income, and family size are significantly positive, with coefficients of 9.360, 9.756, and 3.240, respectively. Column (4) shows the indirect mediated effect, corresponding to $\beta_{1}\times \gamma_{2}$ in Model (3). The indirect mediated effect of models with crop income, livestock income, and family size as intermediate variables is significantly positive, with coefficients of 2.579, 0.772, and 8.714, respectively. Column (5) shows the proportion of the total effect that is mediated, with proportions of 0.216, 0.073, and 0.729 for crop income, livestock income, and family size, respectively.

**Figure 3:**
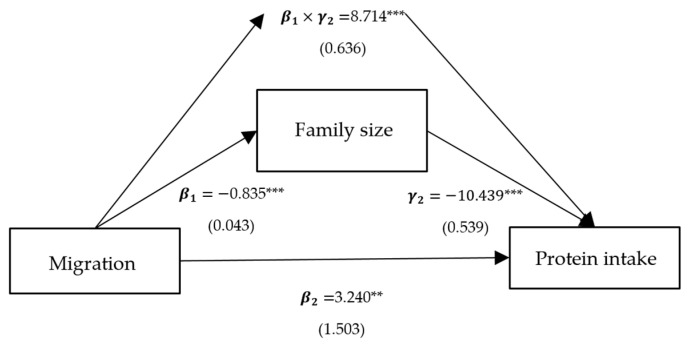
Path analysis of migration, protein intake, and family size. Notes: standardized coefficients are reported with robust standard errors in parentheses. Notes: ***, ** and * indicate significance at 1%, 5% and 10%.

**Figure 4:**
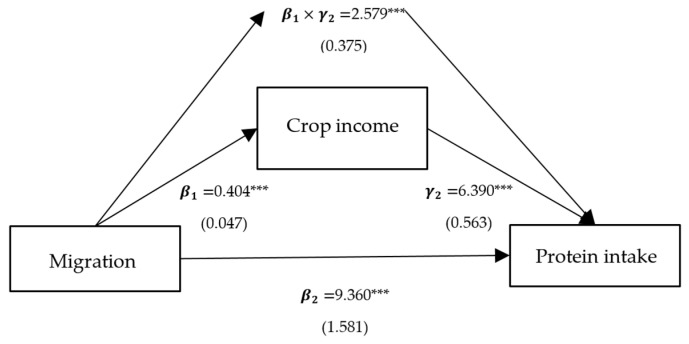
Path analysis of migration, protein intake, and crop income. Notes: standardized coefficients are reported with robust standard errors in parentheses. Notes: ***, ** and * indicate significance at 1%, 5% and 10%.

**Figure 5:**
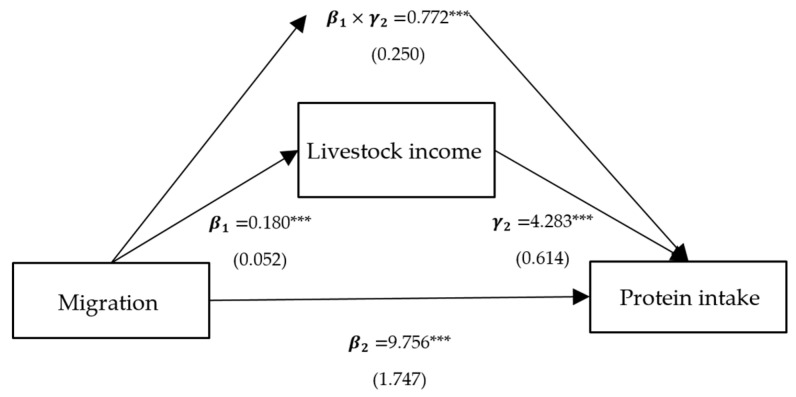
Path analysis of migration, protein intake, and livestock income. Notes: standardized coefficients are reported with robust standard errors in parentheses. Notes: ***, ** and * indicate significance at 1%, 5% and 10%.

**Figure 6:**
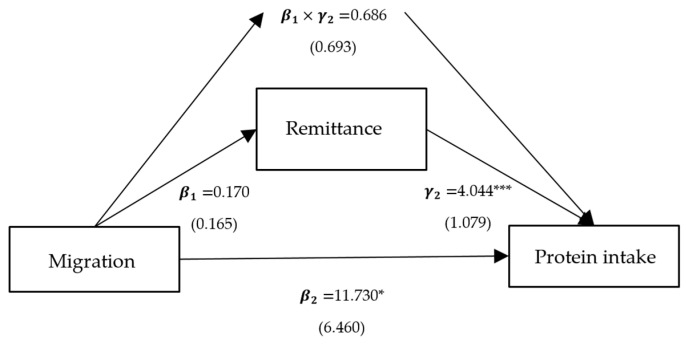
Path analysis of migration, protein intake, and remittance. Notes: standardized coefficients are reported with robust standard errors in parentheses. Notes: ***, ** and * indicate significance at 1%, 5% and 10%.

**Figure 7:**
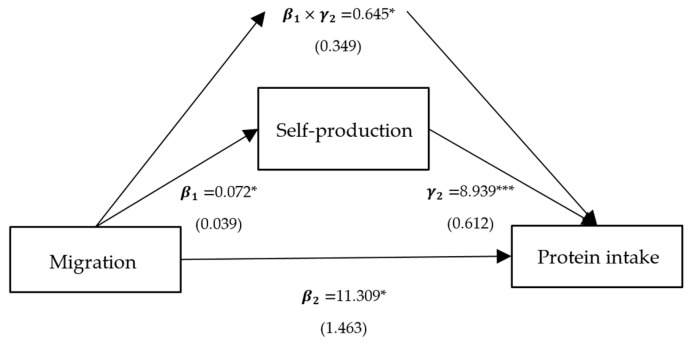
Path analysis of migration, protein intake, and self-production. Notes: standardized coefficients are reported with robust standard errors in parentheses. Notes: ***, ** and * indicate significance at 1%, 5% and 10%.

To sum up, the positive impact of migration on protein intake is predominantly realized through three pathways: crop income, livestock income, and family size. Among these, the mediating effect of migration on protein intake through family size is the largest, at 8.637, accounting for 0.726 of the total effect (Figure 3). Following this is the mediating effect of migration on protein intake through crop income, at 2.577, comprising 0.217 of the total effect (Figure 4). The mediating effect of migration on protein intake through livestock income is the smallest, at 0.772, accounting for 0.074 of the total effect (Figure 5). However, the mediating effects of remittance income (Figure 6) and self-production (Figure 7) are not significant. 

## 4. Discussion

This study investigates the mediating factors between migrant labor and the protein intake of family members left behind in rural areas. Previous studies have predominantly analyzed the relationship between labor migration and dietary nutrition but have seldom quantified the pathways. These studies often utilized relatively small or cross-sectional samples, focusing mainly on the direct income effects of remittances and household characteristics such as the age and gender of the head of household, and dependency ratios [25,28] on food consumption and food insecurity. Our study has advantages in sample selection, covering a longer time span, with a sufficient sample size, focusing on households residing in underdeveloped areas characterized by vulnerable food security status. The study employs a mediating effects model to explore the mechanisms of income structure, self-sufficiency, and household size impacts, providing insights into factors that can improve nutrition in underdeveloped areas, which may be helpful for subsequent research.

Consistent with some previous findings in underdeveloped areas of countries like Bangladesh, Ethiopia, and Vietnam [29,30,31], our results indicate that migrant labor contributes to food and nutritional security. According to the New Economics of Labor Migration (NELM), migration is a decision made by a household, rather than an individual, to minimize risks and stabilize income [28]. Although the impact of migration on food and nutritional security remains contested in studies, for impoverished families, if labor migration can increase per capita disposable income, it can improve the family’s nutritional issues. Per capita disposable income is influenced by both the number of family members and income; migration implies a reduction in family members, the labor loss of which might affect agricultural production. The effects of changes in agricultural income and remittance income on total income are the main sources of contention [29].

Our findings highlight that the reduction in household size has the most significant impact on increasing nutritional intake in rural families due to migration. A smaller household size means fewer people to feed, potentially increasing per capita disposable income and, consequently, food consumption. Besides economic factors, when the number of family members decreases, the main caretaker of the household has more time and energy to care for each member [28], particularly vulnerable groups like children and the elderly, thus helping to enhance the overall nutritional intake.

Migration also enhances household income from crop production and livestock, thereby boosting the protein intake of rural families. Previous studies that discussed how migration could worsen food and nutrition security often cited the inability of remittances to compensate for the decline in agricultural income due to the labor remaining behind as a primary reason [32,33]. This also explains why our conclusions are contrary to this literature. Among them, migration had a greater positive impact on crop income, a trend that may be due to the central focus on crop production in the central and western regions of China, where most of our samples are located. Moreover, migration provides better access to information and more flexibility in liquidity, enabling rural families to overcome credit and risk constraints and thus enhance agricultural productivity [34].

Migration did not significantly increase the remittance income of rural family members, which differs from the majority of the literature [29]. We speculate that this is because migrants invest the remitted income in agricultural production [35,36], which could further explain why our findings indicate that labor migration promotes agricultural income. The mediating effects show that migration does not enhance rural residents’ protein intake through remittance income. These results urgently require further research to understand the relationship between migration and rural welfare and which factors influence the direction of remittances (such as human capital, marketization level, etc.).

Migration also did not significantly increase self-sufficiency, and the mediating effects also show that self-production does not significantly regulate the impact of migration on rural family protein intake, indirectly indicating that agricultural production is primarily for selling agricultural income, and internal consumption has little impact on nutritional intake. Related research emphasizes the shift from subsistence crops to agricultural production diversification, i.e., improving nutritional status by expanding the range of products that families can consume [37].

This study has some limitations. First, the study did not fully explore the long-term impacts of migration on the rural economy and society. Specifically, migration could significantly impact agricultural production structures, social structures, traditions, and community lifestyles, which may indirectly affect the nutritional status in rural areas. Additionally, the study did not cover the phenomenon of reverse migration, triggered by high urban living costs, rural industrial development opportunities, or global events like the COVID-19 pandemic, which could significantly impact rural welfare and nutritional security. These limitations suggest that future research should pay more attention to how migration dynamics affect rural socio-economic structures and how these impacts shape the nutrition and health landscape in rural areas.

## 5. Conclusions

Our study reaffirms the positive impact of migrant labor on the nutritional intake of household members left behind. The mediating effects clearly demonstrate that reducing household size, along with increasing crop and livestock income, significantly enhances household food security. Specifically, the reduction in household size has the largest mediating effect, at 8.714, accounting for 0.729 of the total effect, followed by an increase in crop income at 2.579, contributing 0.216, and an increase in livestock income at 0.772, contributing 0.073.

These findings underscore the importance of a well-balanced household structure, diversified income strategies, and robust marketization in addressing nutritional deficiencies in rural China. We recommend that the government expand healthy eating education and awareness campaigns across rural areas to improve residents’ nutritional knowledge and support healthier dietary choices. Additionally, strengthening market capacity by promoting the direct marketing of local agricultural products can reduce logistics costs, making nutrient-rich foods more affordable and accessible to rural households and simultaneously enhancing farm incomes.

## Figures and Tables

**Table 2:** Descriptive statistics.

	Year	2012 (N = 1368)		2015 (N = 1368)		2018 (N = 1371)	
Variables		Mean	Std. Error	Mean	Std. Error	Mean	Std. Error
Panel A: Household-level core indicators							
Protein intake		74.464	39.394	72.073	54.319	73.989	49.505
Migration (*Mig*)		0.593	0.491	0.569	0.495	0.610	0.488
Migration rate (*Migrate*)		0.225	0.227	0.222	0.234	0.236	0.235
Panel B: Mediated indicators							
Remittance		7.313	1.395	7.418	1.468	7.430	1.347
Crop income		7.449	1.303	7.254	1.329	6.274	1.934
Livestock income		7.041	1.446	7.391	1.590	7.376	1.750
Self-production		3.382	1.238	2.920	1.303	2.823	1.408
Family size		3.446	1.450	3.427	1.543	3.408	1.564
Panel C: Control variables							
Gender of household head		0.907	0.291	0.939	0.239	0.914	0.281
Age of household head		50.659	11.134	52.107	11.127	53.894	11.252
Schooling of household head		6.585	3.665	6.306	3.501	6.440	3.610
Annual per capita income		8.917	0.931	8.975	0.987	9.105	1.142
Proportion of young children		0.060	0.117	0.062	0.123	0.071	0.130
Proportion of school-aged children		0.057	0.123	0.051	0.118	0.040	0.104
Proportion of the elderly		0.127	0.247	0.147	0.270	0.191	0.305
Household wealth index		−0.493	1.86	0.148	1.720	0.908	2.044
Household indebtedness		0.612	0.488	0.605	0.489	0.575	0.494
Distance to market		6.639	8.556	6.708	7.917	6.519	12.555
Land cultivated		5.587	7.356	6.318	14.651	4.499	11.286
Natural shocks		0.842	0.365	0.618	0.486	0.430	0.495
Other shocks		0.700	0.459	0.519	0.500	0.255	0.436

**Table 3:** The effects of per capita protein of rural family.

	(1)	(2)
	*P* *rotein*	*P* *rotein*
*Mig*	8.701 ***	
	(1.476)	
*Migrate*		33.638 ***
		(3.623)
Gender of household head	4.154	4.900 *
	(2.548)	(2.467)
Age of household head	0.261 ***	0.204 **
	(0.077)	(0.076)
Schooling of household head	0.270	0.216
	(0.219)	(0.215)
Annual per capital income	9.223 ***	8.184 ***
	(0.794)	(0.772)
Proportion of young children	−56.445 ***	−50.619 ***
	(5.592)	(5.464)
Proportion of school-aged children	−36.416 ***	−29.766 ***
	(5.411)	(5.429)
Proportion of the elderly	−6.696 *	−7.340 *
	(3.132)	(3.065)
Household wealth index	−1.215 **	−1.086 *
	(0.444)	(0.435)
Household indebtedness	−1.218	−0.922
	(1.561)	(1.536)
Distance market	0.178	0.164
	(0.108)	(0.107)
Land cultivated	0.146	0.176
	(0.123)	(0.125)
Natural shocks	1.055	0.904
	(1.735)	(1.718)
Other shocks	−0.370	−0.108
	(1.534)	(1.523)
Regional fixed effect	Y	Y
Year fixed effect	Y	Y
Cons	−26.050 **	−18.324 *
	(9.309)	(9.156)
N	3831	3831

Notes: ***, **, and * indicate significance at 1%, 5%, and 10%. Heteroscedasticity robust standard errors, clustered at household level, are in parentheses. In this table, the abbreviation “*Mig*” refers to migration (indicating whether a household has members engaged in labor migration), while “*Migrate*” denotes migration rate (the ratio of the number of household members engaged in labor migration to the total household population).

**Table 4:** Results for the mediation model with migration as independent variable, per capita protein of rural family as dependent variable, and the remittance, crop income, livestock income, self-production, and family size as mediator.

Mediator Variable	(1) $\beta_{1}$	(2) $\gamma_{2}$	(3) $\beta_{2}$	(4) $\beta_{1}\times \gamma_{2}$	(5) Proportion of Total Effect That Is Mediated
Remittance	0.170 (0.165)	4.044 *** (1.079)	11.730 * (6.460)	0.686 (0.693)	0.055
Crop income	0.404 *** (0.047)	6.390 *** (0.563)	9.360 *** (1.581)	2.579 *** (0.375)	0.216
Livestock income	0.180 *** (0.052)	4.283 *** (0.614)	9.756 *** (1.747)	0.772 *** (0.250)	0.073
Self-production	0.072 * (0.039)	8.939 *** (0.612)	11.309 * (1.463)	0.645 * (0.349)	0.054
Family size	−0.835 *** (0.043)	−10.439 *** (0.539)	3.240 ** (1.503)	8.714 *** (0.636)	0.729

Notes: ***, ** and * indicate significance at 1%, 5% and 10%. Heteroscedasticity robust standard errors, clustered at household level, in parentheses. All regressions control for characteristics of the household head, household characteristics, and natural characteristics.

## Data Availability

The datasets used in the current study are not publicly available due to the confidential policy but are available from the corresponding author upon reasonable request.
